# High Treatment Retention Rate in HIV-Infected Patients Receiving Antiretroviral Therapy at Two Large HIV Clinics in Hanoi, Vietnam

**DOI:** 10.1371/journal.pone.0139594

**Published:** 2015-09-30

**Authors:** Shoko Matsumoto, Junko Tanuma, Daisuke Mizushima, Ngoc Chi Thi Nguyen, Thanh Thuy Thi Pham, Cuong Duy Do, Tuan Quang Nguyen, Dung Thi Nguyen, Hoai Dung Thi Nguyen, Lam Tien Nguyen, Kinh Van Nguyen, Shinichi Oka

**Affiliations:** 1 AIDS Clinical Center, National Center for Global Health and Medicine, Tokyo, Japan; 2 Graduate School of Public Health, Teikyo University, Tokyo, Japan; 3 Center for AIDS Research, Kumamoto University, Kumamoto, Japan; 4 Department of Infectious Diseases, Bach Mai Hospital, Hanoi, Vietnam; 5 National Hospital of Tropical Diseases, Hanoi, Vietnam; University of Athens, Medical School, GREECE

## Abstract

**Background:**

Loss to follow-up (LTFU) is viewed as a major challenge in improving retention in HIV treatment. In Vietnam, the reasons for disengagement from clinics and the effect of injection drug use (IDU) on LTFU with unknown outcome (true LTFU) are not well known.

**Methods:**

Patients receiving antiretroviral therapy (ART) from two HIV clinics in Hanoi were included in this observational study between 2007 and 2012, and followed up every 6 months until the end of 2013. The reasons for disengagement from the clinic, and ART status during imprisonment were investigated in patients with a history of IDU to identify true LTFU. The retention rate at 6–54 months and true LTFU rate were calculated. Cox proportional hazards regression models were performed to identify factors associated with true LTFU.

**Results:**

There were 1,431 patients, with a follow-up time of 4,371 person-years (median 2.49 years). At the end of the follow-up period, 71 (5.0%) patients died, 79 (5.5%) transferred to other clinics, 16 (1.1%) disengaged from the clinics, and the calculated true LTFU was 45 (3.1%), with 12-month ART retention rate of 95.3% for the entire study population. Imprisonment was the most frequent reason for disengagement from the clinics. True LTFU correlated significantly with low CD4 count and high plasma viral load, but not history of IDU.

**Conclusion:**

Imprisonment is a major cause of disengagement from HIV care among patients with a history of IDU.

## Introduction

Evaluation of loss to follow-up (LTFU) has a considerable impact on estimation of retention in antiretroviral therapy (ART) [1–3]. In Vietnam, previous studies reported that as many as 5–15% of the patients who started ART were lost to follow-up [4–6], although the definitions used for LTFU and duration of follow-up varied. The importance of history of injection drug use (IDU) was highlighted as a strong predictor for LTFU in these studies since IDU is the major route of HIV transmission in Vietnam [4,6]. The authors have suggested that poor adherence, IDU-related stigma, active drug use, and comorbidity [e.g., hepatitis C and tuberculosis (TB)] hamper retention in HIV treatment in people who inject drugs (PWIDs). A wide range of retention rates and various predictors of LTFU have been identified in previous studies, in which LTFU was defined simply by the duration of absence from clinic visits. However, no studies have examined the reasons for disengagement from the clinics and whether the patients discontinued ART at LTFU.

Strict control over illicit drug use has been enforced by the Vietnamese Government and the United Nations Office on Drugs and Crime reported in 2010 that PWIDs accounted for ~30% of the detainees [7]. Once HIV patients on ART are incarcerated in prison, their supporters (e.g., family, partners, or friends) deliver antiretroviral drugs from the HIV clinics to the prison to help the patients continue their treatment. Even patients who do not have such supporters receive ART through the health services in the prison or in nearby HIV clinics when available. Therefore, although study investigators often consider LTFU patients as those who discontinued HIV treatment, imprisoned patients with a history of IDU might not have necessarily discontinued ART.

Existing approaches for measuring LTFU involve patients who do not return to the clinic for a variety of reasons (disengagement from clinic) and those who are lost to follow-up with unknown outcomes (true LTFU) [3,8]. Disengagement from the clinic might be due to patients’ wishes or beliefs, or other social–structural barriers (e.g., transportation, and HIV-related stigma) [3]. The outcomes of true LTFU could be unascertained death, ‘silent’ transfer, or disengagement from the clinic. Under a well-functioning patient tracing system, true LTFU reflects the most hard-to-trace population. Thus, disengagement from the clinic and true LTFU illustrate two different problems. We separately treated these two groups accordingly.

We focused on exploring the reasons for discontinuation of clinical follow-up using a patient tracing system. We assessed whether PWIDs who missed their visits because of imprisonment continued to receive ART. Using this protocol, the present study was designed to identify the retention rate, and factors related to true LTFU among HIV-infected patients on ART in two large HIV outpatient clinics in Hanoi. We also investigated on whether the definition of LTFU affected the impact of IDU on LTFU. Although ART services have been rapidly scaled up in Vietnam [9,10], ART is a life-long treatment. This study evaluated accurate patient outcomes and factors associated with discontinuation of HIV treatment among patients receiving ART.

## Materials and Methods

### Study design and study subjects

We retrospectively reviewed observational cohort data of adult HIV-infected patients (>17 years of age) on ART at the HIV Outpatient Clinics of the Bach Mai Hospital (BMH) and the National Hospital of Tropical Diseases (NHTD). These two institutions have the largest HIV referral clinics in Hanoi. The cumulative numbers of registered HIV patients in these clinics from their opening to November 2013 were 1,408 and 2,879, respectively. Both clinics were supported by the U.S. President’s Emergency Plan for AIDS Relief (PEPFAR) and the patients were provided free ART. We also provided free plasma viral load (pVL) and CD4 count testing once or twice a year after patient enrollment. The subjects for analysis included all those who enrolled in the study at the two clinics between October 1, 2007 and December 31, 2012, and were followed up every 6 months until December 31, 2013. All subjects started ART before enrollment in the study, and were still on ART at enrollment.

### Patient tracing system

The two HIV clinics were operating a patient tracing system under which all patients were contacted by telephone by health professionals when they did not attend their scheduled visits. Both clinics have organized treatment groups, and the patients who missed a scheduled visit were contacted through the group members. Each treatment group comprised 10–30 HIV-infected patients, and medical follow-up schedule, adherence counseling sessions, and other activities were arranged by the groups. Notably at BMH, two peer supporters were selected from among the patients based on their experience and knowledge of HIV. When patients who missed the scheduled visit could not be contacted by telephone, the peer supporters were sent to the patients’ residence. The patient tracing system ascertained the vital status of the patients and the reasons for the missed appointments, and arranged other appointments for medical check-up for the patients. At the end of the follow-up period, the outcomes of all patients and the last dates of clinic visits were entered. When PWIDs discontinued clinic visits because of imprisonment, we assessed whether they were still receiving ART in prison by investigating whether their supporters had visited the clinics to pick up antiretroviral drugs for the patients.

### Measurements

#### Retention

Retention was defined as those patients who were alive and on ART either at the clinic or in prison.

#### True LTFU and disengagement from the clinic

We defined LTFU as patients with unknown outcome, and termed it true-LTFU. In line with the WHO patient monitoring guidelines [11] and other studies related to LTFU conducted in Vietnam [4–6], the following definition was used for true LTFU: patients who stopped visiting the clinics for at least 3 months after their last visit, and did not return by the end of the follow-up period (December 31, 2013). Patients who were transferred to other clinics and those who died during the follow-up period were not included in the true LTFU group. In contrast, patients who ceased to engage in HIV treatment at the clinic (e.g., by their own decision or because of social barriers) were termed as “disengagement from the clinic”, and not included in the true-LTFU group.

#### Independent variables

Independent variables included: name of the clinic, sex, age, history of IDU, latest CD4 cell counts, latest pVL, co-infection with hepatitis B virus (HBV) and hepatitis C virus (HCV), history of TB, and duration of ART at baseline. History of IDU was assessed at enrollment to the cohort. Latest CD4 count and pVL were obtained at the last clinic visit during the follow-up period. For patients who were retained in the cohort, these values were obtained at the most recent visits before December 31, 2013. For patients who were lost to follow-up, these values were obtained at the most recent visits before LTFU. The values for those who died or transferred were obtained in the same manner. History of TB was assessed by whether patients had experienced any TB-related event until the end of the follow-up period. Patients were divided into three groups according to age: <30 years, 30–39 years, and ≥40 years, and into two groups according to latest CD4 count: <350/μl and ≥350/μl. Patients were divided into two groups according to the latest pVL: <500 copies/ml and ≥500 copies/ml. Patients were also divided into three groups according to the duration of ART use at enrollment to the cohort (ART use at baseline): <6 months, 6–11 months, and ≥12 months. Other variables were treated dichotomously.

### Statistical analysis

The retention rates at 6–54 months after enrollment were calculated using the following formula: Number of patients still alive and on ART at the two clinics or in prison at 6–54 months divided by the total number of patients followed up for 6–54 months, including those who died, those lost to follow-up, and those disengaged from the clinic. In line with other studies [4–6], patients who were transferred to other clinics were excluded from the retention analysis because we could not determine whether they were still alive or receiving ART at other clinics. In addition, to identify the approximate retention rate from ART initiation, we also calculated the 6- and 12-month retention rates for those who had started ART within 3 months before enrollment in the study.

Next, we assessed the reasons for discontinuation of clinical follow-up to identify true LTFU patients. The rate of true LTFU based on the characteristics of the participants was then calculated. Finally, Cox proportional hazards regression analysis was used to examine the effect of each independent variable on the incidence of true LTFU. The crude and multivariate adjusted hazard ratio (HR) and 95% confidence interval (CI) were calculated (Model 1 and Model 2). Variables that showed statistical significance (*p*<0.05) in the crude model (Model 1) were used in the multivariate model (Model 2). In these analyses, patients who were transferred to other clinics, those who died during follow-up, and those who were imprisoned but continued ART or had otherwise disengaged from the two clinics were censored at the date of transfer, death, and last clinic visit, respectively.

To investigate whether the definition of LTFU affects the impact of IDU on LTFU, we developed Model 3, in which a broader definition of LTFU was used as the outcome variable. In this model, true LTFU and disengagement from the clinic were treated as LTFU. This allowed comparison of the factors associated with the broader definition of LTFU, as used in previous studies (Model 3) [4,6] to those associated with true LTFU (Model 2). In previously published studies, patients who missed scheduled visits for at least 3 months because of imprisonment, but continued ART in prison, were also included in the definition of LTFU. However, we did not include them in our broader definition of LTFU because of its obvious association with history of IDU.

As a supplementary analysis, we conducted Model 2 after exclusion of HCV co-infection from the explanatory variables, considering its strong correlation with history of IDU [12]. All analyses were performed using SAS version 9.3 (SAS Institute, Cary, NC, USA). All tests were two sided, with significance level set at 5%.

### Ethical statement

The study was approved by the Human Research Ethics Committee of the National Center for Global Health and Medicine (reference: NCGM-G-001074-01), BMH (reference: 40/HDDD), and NHTD (reference:18/HDDD-NDTU). Each participant provided written informed consent for use of their clinical data, and the data were treated anonymously.

## Results

### Study participants

The present analysis involved 1,431 patients (1,057 from NHTD and 374 from BMH); 65% were male and 59% initiated ART within 12 months before enrollment to the study cohort. Eighty-three percent of participants were aged <40 years, and 36% reported a history of IDU. The patient characteristics are presented in Table 1. The total follow-up time was 4,371 person-years [median 2.49 years, interquartile range (IQR) 1.94–4.41].

**Table 1:** Patient characteristics.

	*n*	%	True LTFU n (%)
All	1431	100.0	45 (3.1)
Clinic			
NHTD	1057	73.9	33 (3.1)
BMH	374	26.1	12 (3.2)
Sex			
Male	929	64.9	37 (4.0)
Female	502	35.1	8 (1.6)
Age (years)			
Median (25th, 75th percentile)	32 (29,37)		
<30	410	28.7	20 (4.9)
30–39	775	54.2	19 (2.5)
≥40	246	17.2	6 (2.4)
HIV risk factor			
Non IDU	910	63.6	16 (1.8)
IDU	521	36.4	29 (5.6)
Latest CD4 count (/μl)			
Median (25th, 75th percentile)	371 (250, 512)		
<350	655	45.8	32 (4.9)
≥350	776	54.2	13 (1.7)
Latest plasma viral load (copies/ml)			
<500	1354	94.6	32 (2.4)
≥500	76	5.3	13 (17.1)
Missing	1	0.1	0 (0.0)
HBV co-infection^a^			
Yes	187	13.1	5 (2.7)
No	1226	85.7	39 (3.2)
Missing	18	1.3	1 (5.6)
HCV co-infection^b^			
Yes	617	43.1	29 (4.7)
No	677	47.3	12 (1.8)
Missing	137	9.6	4 (3.0)
History of tuberculosis			
Yes	242	16.9	9 (3.8)
No	1189	83.1	36 (3.0)
Duration of ART at baseline (months)			
Median (25th, 75th percentile)	5 (2, 9)		
<6	460	32.2	14 (3.0)
6–11	380	26.6	9 (2.4)
≥12	585	40.9	21 (3.6)
Missing	6	0.4	1 (16.7)

*n*; number of participants,

LTFU; loss to follow-up,

NHTD; National Hospital of Tropical Diseases,

BMH; Bach Mai Hospital,

IDU; injection drug use

^a^HBV co-infection was assessed by HBV antigen positivity at registration

^b^HCV co-infection was assessed by HCV antibody positivity at registration.

### Retention rates

At the end of the follow-up period, 71 (5.0%) patients died, 79 (5.5%) were transferred to other clinics, 16 (1.1%) disengaged from the two clinics, and 45 (3.1%) were true LTFU, while the remainder (1220, 85.3%), including eight patients in prison, continued their ART.

**Fig 1:**
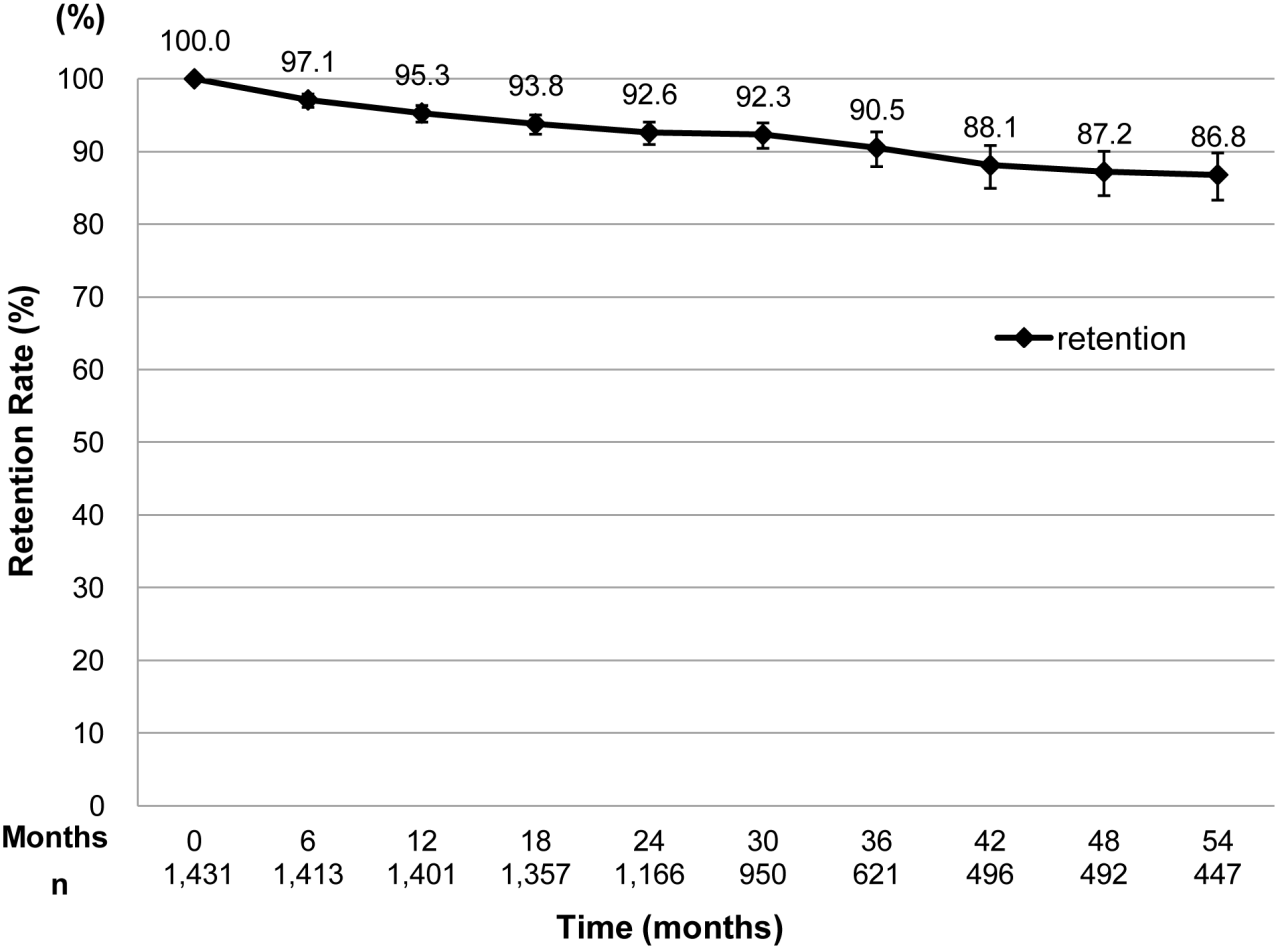
Retention rate for ART at 6–54 months after study enrollment. The retention rate was calculated by dividing the number of patients who were still alive and on ART at 6–54 months by the total number of patients who had been followed up for 6–54 months, including those who died, those lost to follow-up, and those who disengaged from the clinic. Patients who were transferred to other clinics were excluded. Months; months after enrollment, n: number of the subjects for analysis,—;95%CI.

Fig 1 shows the retention rate at 6–54 months after study enrollment. The retention rates at 6- and 12-months were relatively high [97.1% (95%CI: 96.1–97.9%, and 95.3% (95%CI: 94.1–96.3%), respectively]. A total of 178 patients had started ART within 3 months before enrollment. The 6- and 12-month retention rates for the latter group were 96.1% (95% CI: 92.1–98.4%) and 93.2% (88.5–96.5%), respectively (data not shown).

### Rate of disengagement from clinic

Sixteen patients (1.1%) disengaged from the two clinics (Table 2). Eleven (68.8%) disengaged because of imprisonment, but whether they continued to receive ART in prison was not confirmed since none of the relatives/friends visited the clinic to pick up antiretroviral drugs.

**Table 2:** Reasons for disengagement from the two clinics.

	*n*	%
Imprisonment	11	68.8
Refusal of ART	2	12.5
Self adoption of antiretroviral drug	1	6.3
Moved overseas	1	6.3
Other	1	6.3

*n*; number of participants.

### True LTFU rate

At the end of the study, 45 patients (3.1%) were identified as true LTFU, with an incidence of 1.03 per 100 person-years. The median time from study enrollment to true LTFU was 401 days (IQR 1–587 days). Fifteen patients (33.3%) were lost to follow-up after their first visit to the study center and assigned one day of person-time. Table 1 shows the proportions of true LTFU based on the clinical characteristics of participants.

The results of Cox proportional hazards regression models (Models 1–3) are shown in Table 3. In Models 1 and 2, true LTFU was the outcome variable, while in Model 3, the broader definition of LTFU was used (disengagement from clinic was included in the definition of LTFU). In Model 2, latest CD4 count <350/μl (HR = 2.69, 95% CI: 1.30–5.57) and latest pVL ≥500 copies/ml (HR = 5.34, 95% CI: 2.53–11.26) correlated significantly with higher likelihood of true LTFU. In contrast, in Model 3, not only latest CD4 count <350/μl (HR = 2.10, 95% CI: 1.15–3.82) and latest pVL ≥500 copies/ml (HR = 5.52, 95% CI: 2.87–10.62), but also history of IDU (HR = 2.49, 95% CI: 1.09–5.68) correlated significantly with the broader definition of LTFU.

**Table 3:** Results of Cox proportional hazard regression analysis.

	true LTFU						true LTFU + disengagement from clinic		
	Unadjusted			Adjusted			Adjusted		
Outcome	Model 1			Model 2			Model 3		
	(*n* = 1431)			(*n* = 1285)			(n = 1293)		
	HR	95%CI	*p*-value	HR	95%CI	*p*-value	HR	95%CI	*p*-value
Clinic									
NHTD	0.72	0.36–1.43	0.34						
BMH	1.00								
Sex									
Male	2.57	1.20–5.53	0.02	1.23	0.43–3.51	0.69	1.23	0.50–3.03	0.65
Female	1.00			1.00			1.00		
Age (years)									
<30	1.93	1.03–3.62	0.04	1.85	0.94–3.62	0.07	1.44	0.80–2.59	0.23
30–39	1			1.00			1.00		
≥40	1.08	0.43–2.70	0.87	1.16	0.41–3.24	0.78	1.67	0.78–3.58	0.19
HIV risk factor									
Non IDU	1.00			1.00			1.00		
IDU	3.23	1.75–5.94	< .001	2.10	0.82–5.39	0.12	2.49	1.09–5.68	0.03
Latest CD4 count (/μl)									
<350	3.75	1.95–7.21	< .001	2.69	1.30–5.57	0.01	2.10	1.15–3.82	0.02
≥350	1.00			1.00			1.00		
Latest plasma viral load (copies/ml)									
<500	1.00			1.00			1.00		
≥500	11.57	6.02–22.24	< .001	5.34	2.53–11.26	< .001	5.52	2.87–10.62	< .001
HBV co-infection^a^									
Yes	0.84	0.33–2.14	0.72						
No	1.00								
HCV co-infection^b^									
Yes	2.75	1.41–5.39	< .01	1.26	0.51–3.07	0.62	1.20	0.56–2.61	0.64
No	1.00			1.00			1.00		
History of tuberculosis									
Yes	1.26	0.61–2.61	0.54						
No	1.00								
Duration of ART use at baseline (months)									
<6	1.31	0.57–3.03	0.53						
6–11	1.00								
≥12	1.32	0.60–2.90	0.49						

Model 1; crude model;

true LTFU was used as the outcome variable,

Model 2; multivariate adjusted model;

true LTFU was used as the outcome variable;

variables that were statistically significant in Model 1 with *p*<0.05 were used,

Model 3; multivariate adjusted model; a broad definition of LTFU (true LTFU + disengagement from clinic) was used as the outcome variable.

*n*; number of participants,

95%CI; 95% confidence interval,

HR; hazard ratio,

NHTD; National Hospital of Tropical Diseases,

BMH; Bach Mai Hospital,

IDU; injection drug use.

^a^HBV co-infection was assessed by HBV antigen positivity at registration

^b^HCV co-infection was assessed by HCV antibody positivity at registration

Exclusion of HCV co-infection from the variables in Model 2 did not change the direction of effect of each variable, relative to the original model, but the association between the broader definition of LTFU and history of IDU became more significant with *p* = 0.04 (data not shown).

## Discussion

Previous studies on retention rate and LTFU in HIV treatment and care in Vietnam used the duration of absence from the clinic as the definition of LTFU, but the reasons for the absence were not fully assessed. In the present study, we identified patients who continued to receive ART in prison, patients who disengaged from the clinic for reasons (disengagement from clinic), and those who were lost to follow-up with unknown outcomes (true LTFU), using a patient tracing system that was operated by two large HIV outpatient clinics in Hanoi, Vietnam. The collected data showed a high retention rate at 12 months of 95.3% and low true LTFU rate of 3.1% at the end of follow-up. Although imprisonment was found to be the major cause of disengagement from the clinic, history of IDU was not associated with true LTFU.

We found a higher 12-month retention rate compared with that of 80–84% in previous studies conducted in Vietnam [4–6,9]. However, it is not possible to make a simple comparison between our results and those of the previous studies because ART status of the study participants at baseline differed among the studies. The previous studies followed up patients from initiation of ART. In contrast, our study participants had already initiated ART before the start of our study. Considering the unstable medical condition and various challenges following ART initiation (e.g., fear of side effects, pill burden, and denial of disease), this discrepancy might be one reason for the difference in retention between the present study and earlier investigations from Vietnam. However, the retention rate was still high when it was calculated based on data only from patients who started ART within 3 months before enrollment. Furthermore, some studies from other Asian countries calculated LTFU rate among HIV-infected patients who had an experience of ART before the start of their study. They actually found higher LTFU rates than our rate of 1.03 per 100 person-years. One study using data from 18 sites in the Asia–Pacific region reported an LTFU rate of 21.4 per 100 person-years, although around half of them had temporal LTFU [13]. Another study from Japan found an LTFU rate of 2.49 per 100 person-years [14]. Although the definition of LTFU varied between our study and previous studies, it is reasonable to conclude that the rate of retention in our study is relatively high.

Another reason for the high retention could be the characteristics of our study sites. A previous observational cohort study found higher retention rate and lower LTFU rate in clinics at tertiary level facilities compared with clinics at district level facilities [5]. This might have been due to a better patient tracing system or a better allocation of resources and technical support in tertiary level clinics. Furthermore, the free services of ART or testing of pVL and CD4 count could have worked as an incentive and enhanced retention of patients in the two selected clinics.

Finally, we believe that the patient tracing system used in our study could have been a major contributor to the high retention rate. The system is not driven only by health professionals at the clinics, but also by treatment group members or peer supporters. Such a system could have been more effective for Vietnamese patients with HIV experiencing deep-rooted stigma and discrimination [15,16] and having little social interaction outside the family [16,17]. Various network-based social supports could perhaps encourage people living with HIV to maintain treatment [18–20]. Further studies are needed to confirm the role of social support in retention.

What are the factors associated with true LTFU? Model 2 tested in our study identified lower latest CD4 count and higher latest pVL to correlate significantly with higher incidence of true LTFU. This finding implies that patients were too ill to visit the clinics or that the patients had already died, but neither circumstance was reported to the two clinics. Using the patient tracing system, patients’ deaths were mostly reported by families. However, not all patients lived with their families and it is not uncommon for patients to often change their contact details for fear of disclosure of their HIV status [21]. Therefore, it is difficult to trace patient deaths especially in HIV-infected population.

What is the impact of IDU on LTFU? Model 3 used the broader definition of LTFU, that is, similar to that used in previous studies [4,6]. We compared the factors associated with the broader definition of LTFU identified by Model 3 with those associated with true LTFU by Model 2. Apart from lower latest CD4 count and higher latest pVL, history of IDU was only associated with the broader definition of LTFU. Furthermore, imprisonment was the most frequent reason for disengagement from the clinic. The results imply that the strong association of history of IDU with LTFU reported in previous studies could have been due to the inclusion in the definition of LTFU of patients who disengaged from the clinic and those who continued ART in prison. Although the above previous studies indicated that IDU-related factors (e.g., poor adherence, IDU-related stigma, active drug use, or comorbidity) could hamper retention of patients with IDU history, our data imply that such patients simply lose an option for continuation of medical follow-up because of imprisonment. A weak association between history of IDU and true LTFU was present when HCV co-infection was excluded from the analysis. This might be because history of IDU and HCV co-infection are associated with each other, but history of IDU has a greater association with true LTFU. Nevertheless, we cannot draw any firm conclusion about the impact of IDU history and HCV co-infection on LTFU from our analyses because of the small number of patients who had either a history of IDU or HCV co-infection. In-depth analysis is needed to determine which aspects of history of IDU and HCV co-infection contributes to LTFU in future studies.

It is important to determine whether the difference in the definition of LTFU affects estimation of retention and LTFU rates and predictors of LTFU. Better evaluation of patient outcomes and identification of the reasons for disengagement from HIV programs requires the implementation of sensitive patient tracing systems. Furthermore, although the government has been making efforts to improve HIV services in prison [7], there is still a need to strengthen coordination and build extensive networks among various health facilities and the prison to ensure continuation of treatment and to monitor patient outcome. Some existing studies have reported a similar adherence to ART among PWIDs compared with non-PWIDs if provided adequate support [22, 23]. Interventions at individual and at health system level could increase retention among HIV population including those with a history of IDU history [24].

We investigated retention and the factors associated with true LTFU in Vietnam using data obtained through a patient tracing system. However, the present study had some limitations. First, our study participants were limited to patients receiving ART for a certain time before enrollment in the cohort. Nevertheless, our study is still important because HIV-infected patients require life-long ART, and it is also meaningful for future studies to investigate retention and LTFU among those who just started ART using the same protocol as in the present study. Second, the HIV epidemic in Vietnam is to a large extent limited to PWIDs, accounting for 44% of the reported HIV infections [25]. In contrast, only 36% of the study participants declared a history of IDU at study enrollment. It is not uncommon that PWIDs do not disclose their history of drug use to health professionals because of concern that their HIV-positive status could be reported to their family or the police. This often happens when they are current drug-users. The impact of IDU on true LTFU might have been reduced by such underreporting. Third, there is a geographical difference in HIV prevalence and risk-behavior characteristics in Vietnam [9,26]. The present results cannot be generalized to other areas of Vietnam. Fourth, we did not collect data on social–structural factors (e.g., socio-economic status, transportation, and family structure). Considering the impact of such factors on patient outcome [27–29], future research needs to include such factors in the analysis. Fifth, we assessed whether PWIDs were receiving ART in prison by investigating whether their supporters had visited the clinics to pick up antiretroviral drugs for the patients. This could have biased the results. However, we believe that this was a good proxy for continuation of ART in prison because we found that pVL and CD4 count among patients who revisited the clinic after release were not worsened during imprisonment. The prisoners could continue treatment with support from their family or friends. Finally, the study participants were not randomly selected from the patients at study location. Random sampling is recommended in future studies.

In conclusion, the use of a patient tracing system allowed the identification of high retention and low true LTFU in HIV patients on ART in Hanoi, Vietnam. Imprisonment is potentially the major cause of disengagement from the clinic among patients with a history of IDU.
